# Diachronic Change within the Still Bay at Blombos Cave, South Africa

**DOI:** 10.1371/journal.pone.0132428

**Published:** 2015-07-02

**Authors:** Will Archer, Philipp Gunz, Karen L. van Niekerk, Christopher S. Henshilwood, Shannon P. McPherron

**Affiliations:** 1 Department of Human Evolution, Max Planck Institute for Evolutionary Anthropology, Deutscher Platz 6, D-04103 Leipzig, Germany; 2 Institute for Archaeology, History, Culture and Religious Studies, University of Bergen, Øysteinsgate 3, N-5007 Bergen, Norway; 3 Institute for Human Evolution, University of the Witwatersrand, Johannesburg, South Africa; Universidade do Algarve, PORTUGAL

## Abstract

Characteristically shaped bifacial points are stone artefacts with which the Middle Stone Age Still Bay techno-complex in Southern Africa is identified. Traditional approaches such as *chaîne opératoire* and two-dimensional metrics in combination with attribute analyses have been used to analyse variability within Still Bay point assemblages. Here we develop a protocol to extract and analyse high resolution 3-dimensional geometric morphometric information about Still Bay point morphology. We also investigate ways in which the independent variables of time, raw-material and tool size may be driving patterns of shape variation in the Blombos Cave point assemblage. We demonstrate that at a single, stratified Still Bay site points undergo significant modal changes in tool morphology and standardization. Our results caution against (1) treatment of the Still Bay as a static technological entity and (2) drawing demographic inferences stemming from grouping Still Bay point collections within the same cultural label.

## Introduction

Still Bay points characterize the first of two stratigraphically succeeding, technologically innovative phases within the Southern African Middle Stone Age period (hereafter “the MSA”). The Still Bay and the Howiesons Poort are associated with an array of material markers interpreted as representing cultural complexity in early behaviourally modern humans (hereafter “markers”) [1–7]. These include raw-material heat-treatment, worked bone, ornaments, systematic use of minerals such as ochre, various socially mediated material indicators of abstract thought and complex—potentially multi-component—lithic technologies [1,2,8–15].

Much consideration has been devoted to the initial presence, spatial distribution and disappearance of these markers across southern Africa [16–19]. In this context, the Still Bay and Howiesons Poort are often treated as relatively stable culture-historical entities whose presence in the record is interpreted as mapping different instances of a common demographic network and associated tool-making ‘traditions’ [20–24].

Interestingly—in clear contrast to the Later Stone Age record of this part of Africa [25–29]—much less consideration has been paid to diachronic and synchronic tendencies *within* these MSA phases, such as variation in specific markers or lack thereof through time and across space [5,30–32]. This may be a consequence of the paucity of Still Bay or Howiesons Poort occurrences that exhibit the combination of (1) sufficiently large samples of individual markers and (2) dated sequences that bracket substantial periods of time against which potential evolutionary trends can be modelled and interpreted.

Here we focus on variation in a single marker, Still Bay bifacial points, the *fossile directeur* of the Still Bay phase [1,22,33–35] using the statistical shape analysis tools of geometric morphometrics [36,37]. Three dimensional geometric morphometric techniques have not yet been used to analyse shape variability in bifacial points. The aim of this paper is thus twofold: (1) we outline a protocol for analysing shape differences among bifacial points using three-dimensional geometric morphometrics specifically to (2) investigate patterns of variation in bifacial points at the multi-layered and well-dated Still Bay sequence of Blombos Cave, South Africa.

We consider whether early modern behaviours associated with the production of Still Bay points varied systematically through time or to what degree the onset and proliferation of Still Bay point production was relatively stable. Our analysed sample comprises 119 provenienced, complete, bifacial points produced across a sequence potentially spanning 10 ky and providing a unique window into fine-scale early modern behavioural variation.

Our analyses incorporate 510 three-dimensional geometrically correspondent measurements (landmarks) on each specimen to demonstrate that through time Still Bay bifacial points underwent subtle but directional changes in morphology and standardization. We approach the analysis of our dataset with a series of expectations about prospective patterning, structured loosely on Isaac’s [38] *Method of Residuals* approach to understanding lithic variability. This entails interpreting variation using a stepwise method, selecting the most parsimonious independent variable that could explain a documented pattern first. Once the variation associated with the most parsimonious variable has been investigated, the residual variance is interpreted against the second most parsimonious variable, and so on. Isaac provides a hierarchical sequence that can be followed in this regard. We conduct analyses that enable us to interpret the effects of time, raw-material availability and tool size—including their respective interactions—on patterning in bifacial point variability.

## Background

### Chrono-cultural framework

The term “Still Bay” is generally used to refer to a Southern African Middle Stone Age “techno-tradition” [18,22,23,39–41]. The term was initially suggested to describe a stone tool industry characterized by bifacial points that were purportedly similar in form to “Solutrean” points from eastern France [33,42]. The *fossile directeur* of the techno-tradition was initially recognised as the “laurel-leaf” shaped bifacially flaked stone point [43]. In the early days of characterizing the industry, there seems to have been more debate regarding which point morphologies could reliably be labelled as Still Bay than there is today. For example, contrary to Goodwin and van Riet Lowe [33], Heese initially suggested that the “oak-leaf” shaped point—a morphological variant of the “laurel-leaf” shaped point—should *not* be included in the definition of the Still Bay. He later drastically broadened his perception of what the term Still Bay encompassed, and suggested that the Still Bay also included a number of other previously distinguished industries, including the Howiesons Poort [43].

**Fig 1:**
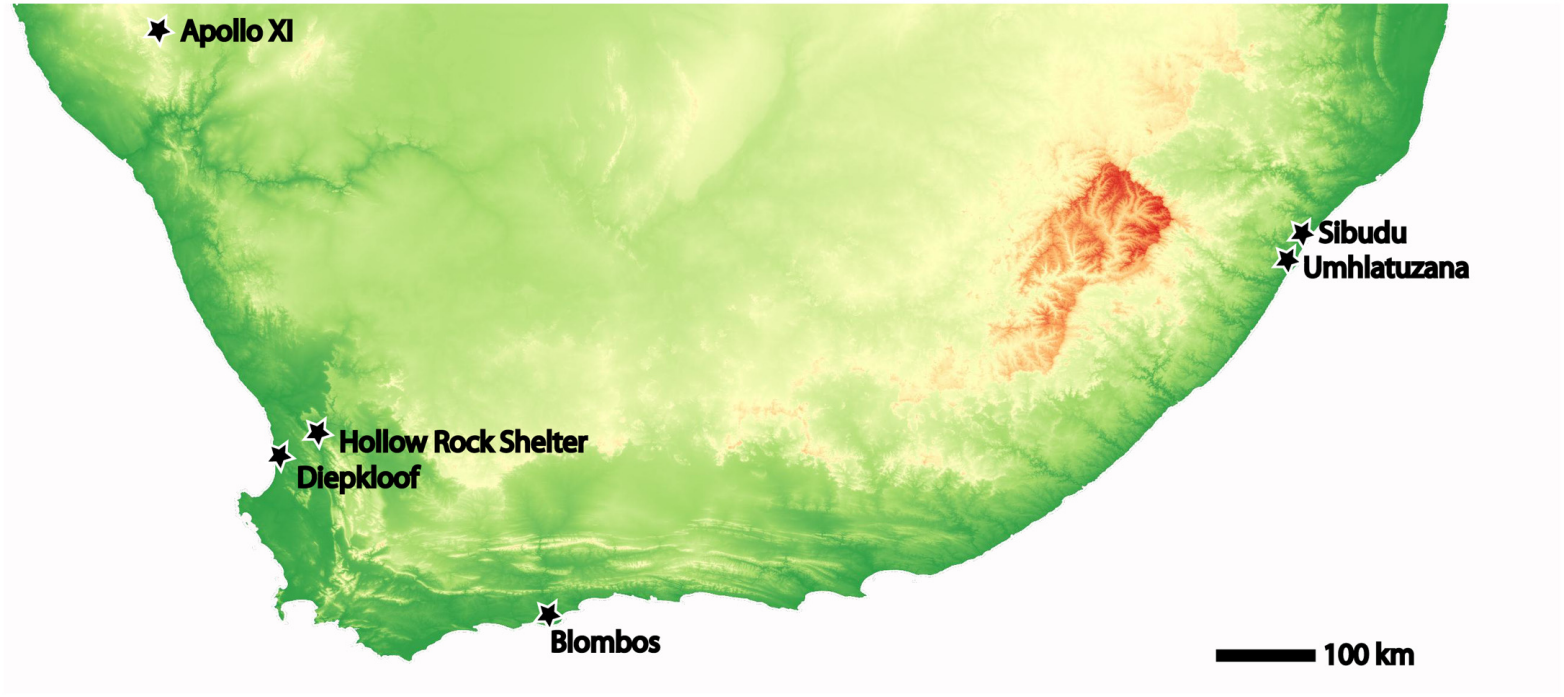
Sites mentioned in the text.

Today a range of dated Still Bay sites exist across southern Africa, including Blombos, Diepkloof Rockshelter, Apollo 11, Hollow Rock Shelter, Sibudu Cave and Umhlatuzana [22,35,40,44–48](Fig 1). Recent excavations of Still Bay sites with good context has widened the definition of the term to associate it also with material evidence for symbolically mediated behaviours such as engraved ochre, shell beads and bone tools [9,10,41]. The term generally encompasses assemblages within a specific Middle Stone Age chronofacies, although a small number of sites exist within the period traditionally associated with the Still Bay that do not in fact have Still Bay assemblages [21,49].

The presence of characteristically shaped bifacial points is still the single criterion by which instances of the techno-tradition are identified in the archaeological record. Today the label “Still Bay” is used largely as a heuristic tool to convey the possibility that one or more groups—or sub-groups within a trans-regional network—manufactured stone points by means of a bifacial shaping tradition to create symmetrical foliate or lanceolate forms through both direct percussion and occasionally pressure flaking techniques [23,50]. Interestingly, the proportions of complete bifacial points that meet these classic design criteria within identified Still Bay localities are low (e.g. [34]).

Further problems arise when one considers that the Middle Stone Age interval the Still Bay tradition potentially spans is widening [47], and that instances of seemingly regionally distinctive point morphologies have already been identified in different parts of Southern Africa [34,46,51]. Additionally, bifacial point production strategies were practiced in various parts of Africa at different stages in the MSA making it implausible that these occurrences on a continental scale relate to a single process of common descent.

The chronological and spatial structure of Still Bay occurrences is also contentious with regard to (1) applied statistical parameters within methodologies used to date them and (2) substantial divergence between chronologies proposed for single localities [19,40,44,45,52]. Consequently, inter-regional groupings of Still Bay bifacial points within the same techno-tradition entails choosing one chronology over another [16,19].

Such grouping remains heuristically useful for documenting broad spatial distributions in Middle Stone Age bifacial points. Yet second order inferences such as identifying locations of origin and processes of cultural transmission are contingent on establishing a common scale of contemporaneity between spatially disparate bifacial point assemblages. Without (1) refinement of the set of specific morphological criteria that link these assemblages, including the data demonstrating that these links exist, and (2) clarification of their chronological association, the demographic significance of grouping them has to remain speculative.

Thus, retaining the notion that the Still Bay represents a demographically meaningful chronofacies entails broadening its definition to span longer periods and wider ranges of forms than the label previously encompassed. This practice also tends to underplay diachronic and synchronic variation in Still Bay point design that may be behaviourally meaningful.

In the present research we select an individual sequence to investigate the structure of variability within the Still Bay in a context where chrono-stratigraphic relationships between individual, provenienced points are sound.

### Still Bay bifacial point technology

The term *bifacial technology* groups the full spectrum of biface types—of which bifacial points are one example—that share a series of binary morphological design parameters such as tip to base opposition and plano-convex versus bi-convex hierarchy in opposing surfaces [24,53]. All biface types result from the application of bifacial knapping strategies, either singularly sequential (knapping face “A” continuously, and then face “B” continuously) or alternating (alternating between knapping face “A” and face “B”), that affect aspects of tool symmetry [54]. These and other common themes amongst bifacial technologies suggest that central features of biface shape likely vary similarly in response to core sets of behavioural drivers.

Technological approaches to Still Bay bifacial point technologies can be categorised broadly in the following ways:

1. Those that reconstruct the production, maintenance and recycling episodes represented in assemblages *a posteriori* and/or through replication [5,34,35].
2. Two-dimensional metrics in combination with attribute identification and analyses [22,46,55].

An alternative—not yet applied to Still Bay collections—is(3) Geometric morphometric and three-dimensional geometric morphometric analyses of bifacial point shape variability [56–59].

A major problem with applying geometric morphometric techniques to analyse bifacial tools is the paucity of available homologous landmarks. Previous studies of “non-Still Bay” bifacial industries have attempted to develop ways of getting around this problem in a two dimensional geometric morphometric analysis context, generally using digital images of the specimens to place landmarks. However, these methodologies rely on the stone tools being relatively flat and not being substantially variable in the bifacial axis [60,61]. For instance Buchannan and Collard [60] and Monnier and McNulty [62] both use landmarks at the base and tip to orient each specimen, then use digital “combs” on photographs of each specimen to superimpose evenly spaced semilandmarks along each edge of each tool. The edge configurations are then subjected to geometric morphometric analyses. Buchannan and Collard [63]employ a slight variation on the edge landmarking method by using combinations of *inter*-landmark linear measurements in association with a set of qualitative descriptors of point morphology, as opposed to analyses of the edge landmark configurations themselves.

Geometric morphometric methods based on digital images of points rely solely on configurations of landmarks derived from the tool edges. In this sense, much shape information associated with three dimensional aspects of the tools two faces is potentially lost.

Previous analyses of Still Bay points at Blombos focused on documenting different stages in bifacial point production through a replication program and the subsequent classification of archaeological points in accordance with the constructed experimental scheme [34]. Additionally, what specific strategies were applied within these stages in terms of how opposing faces were organized and how surface hierarchy influenced final product morphology was also a focus [5,34].

In more general terms, analyses of handaxes demonstrate that dominant components of biface shape variability are often associated with trajectories of tool manufacture and their subsequent reduction [64–69] as well as variability in raw-materials [70,71]. The latter variability has a measureable effect on both (1) shape associated with initial design, and (2) patterns of shape change that points undergo when they are manufactured, maintained and reduced. Similarly, substantial amounts of variation in bifacial point morphology have been shown to be underpinned by maintenance behaviours that are indicative of longitudinal as opposed to lateral bifacial modifications [56–58,72–74].

## Materials

The analysed assemblage comprises a collection of 119 complete, provenienced bifacial points from Blombos cave. The analyses of shape presented here rely on individual points being oriented along two axes at the outset. Firstly, on the bilateral axis (the tip/base opposition and left/right axis) and secondly, on the bifacial axis (the plano-convex opposition of the two faces: see description in Methods). For 26 bifacial points the tip and base could not be distinguished from one another meaning that the possibility of an inverted orientation could not be excluded. These artefacts were removed from the analyses of shape because if certain points were oriented incorrectly with base instead of tip upwards this could blur the documentation of shape variation in general and specifically obscure those aspects associated with tool symmetry. Consequently, the assemblage level descriptive statistics have a sample size of 119 whereas analyses of point shape associated with raw-material variability have a sample size of 93.

### Stratigraphic description

**Fig 2:**
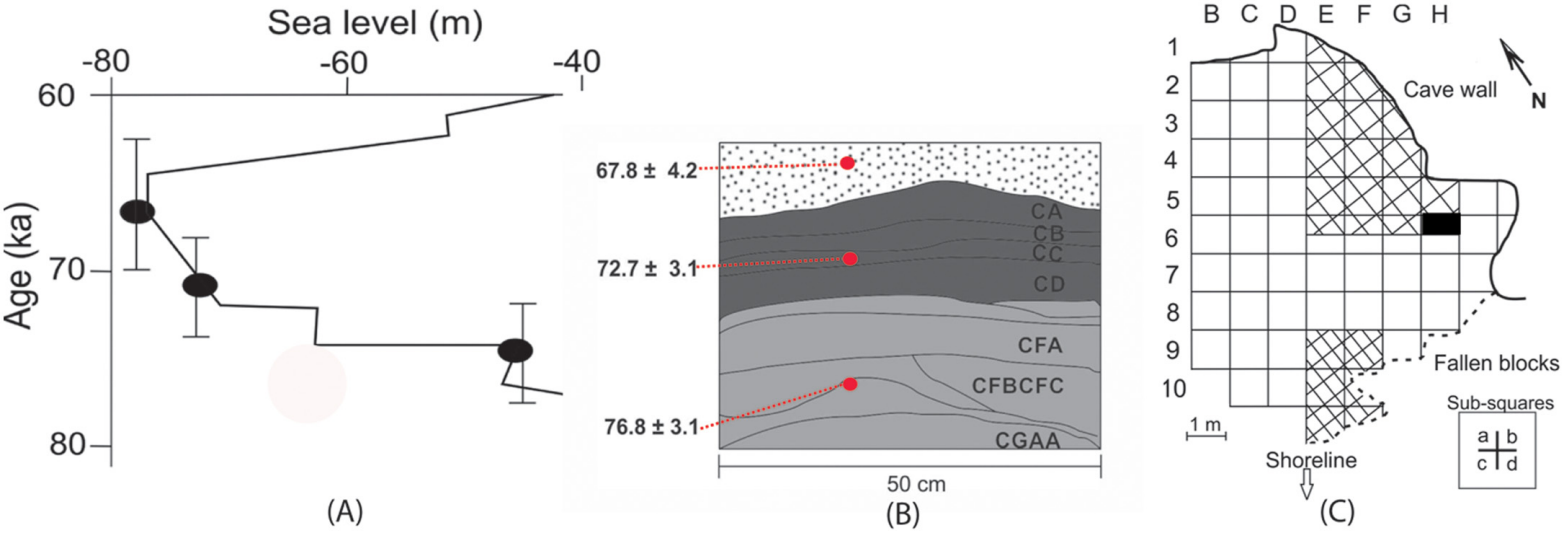
(A) Southern African sea-level curve covering the time period discussed in the text (redrawn from [111]). (B) Stratigraphy of Blombos Cave encompassing the layers discussed in the text. The section is a smaller portion of the section in square H6. The ages are from [77](C) Layout of the shelter and excavated areas (excavations are cross-hatched).

The analysed bifacial points come from an excavation of 66.5, 50 cm by 50 cm sub-squares, from two non-contiguous areas, eight squares of which are located outside of the cave drip line [34] (Fig 2).

The bifacial point bearing deposits from the site are broadly distinguished by chronological phases M1 and M2. Each phase is subdivided into sequential stratigraphic layers that exhibit differences in sediment compaction, composition, colour and cultural materials [2]. The M1 phase comprises the upper layers and constitutes the overall bulk of the Still Bay deposits, including layers CA, CB, CC, CD as well as CE, and is subdivided into M1 Upper (M1a) and Lower (M1b) sub-phases based on clear distinctions in the frequency of small lithic debris and roof-spall [2].

The majority of the analysed points come from the M1 layers, with only a very small proportion (6%) clearly associated with the upper M2 layers. The points are grouped here in accordance with individual layer information for the M1 Upper layers CA, CB, CC, CD and CE. Layer CE had only two points and is an ephemeral horizon. These two layers, CE and CD, were therefore lumped together for statistical purposes. The upper M2 phase layers did not contain more than three points per layer, so the seven complete points from the M2 phase were analysed as a single chronological unit (referred to here as M2) (Table 1).

**Table 1:** Points with individual layer provenience information within the collection that were used in the analyses that uses chronology as a predictor.

*Layer*	*Sub-Phase*	Silcrete	Quartzite	Quartz	Unidentified	Totals
*CA*	*M1 Upper*	6	5	3	1	15
*CB*	*M1 Upper*	7	3	1	0	11
*CC*	*M1 Lower*	8	2	2	1	13
*CD and CE*	*M1 Lower*	23	1	2	0	26
*M2 phase*	*M2*	5	2	0	0	7

Some layers were redefined by the excavators after 1997 when slumped faulted deposits and consequent admixture between Later and Middle Stone Age materials was identified in the rear of the cave [2]. Additionally the complete point collection comes from two laterally disconnected excavations. For these reasons, only points that are unequivocally associated with individual layers within the sequence identified in Henshilwood et al. [2] were used for the analyses that use chronology as an independent variable (n = 72) (Fig 2 represents the relevant portion of this section).

Two of the OSL ages associated with the Still Bay at Blombos come from layers within the sequence, whereas one age comes from sterile, aeolian deposits just overlying the point bearing horizons (Fig 2). The latter deposit yielded an age of 67.8±4.2 ka. An age of 72.7±3.1ka was measured for the M1 point bearing layers (layer “CC”) and an age of 76.8 ±3.1 ka was measured for the lower point bearing layers that are technically in the upper M2 phase (layer CFD) [9,75–77].

These ages suggest that the site was occupied by Still Bay point producing groups by the end of MIS5a, who either continuously or discontinuously occupied the shelter up until the start of MIS4. Recent studies have identified significant change through time in other aspects of early human material culture in this very same Still Bay sequence [31]. Their results suggest that occupation of the site through the M1 phase meant populations were subjected to significantly varying environmental and climatic conditions, which were reflected in marked variations in shell-bead collection and stringing strategies through time ([31]:514). A prediction of the present study is that aspects of stone tool material culture may also have been subjected to these changing environmental conditions over time.

### Raw-materials

Both the Still Bay and the Howiesons Poort in general are characterised by a relative increase in the use of fine-grained, often exotic, raw-materials [5,78]. 67.2% (80 points) of the analysed Blombos assemblage were made from silcrete, which is a material available from primary source outcrops in excess of 20 km from the site [34,79]. However, secondary sources of silcrete may have been available locally in the form of beach cobbles exposed at lower than present day sea levels [80]. Quartzite (22 points: 18.4%) and quartz (15 points: 12.6%) make up the proportion of points produced on local raw-materials. These frequencies largely mirror the frequencies identified by Villa et al. [34] for the collection that includes point fragments (silcrete: 71.7%, quartzite: 15.1%, quartz: 12.9%). In addition, two complete points were produced on crypto-crystalline raw-materials from unknown sources. All individual layers are dominated by silcrete, and the small sample of points from the M2 phase is the only unit that does not contain any complete quartz points.

## Methods

Our objective in this study is to identify MSA hominin behaviours that underpin highly resolved aspects of bifacial point shape at Blombos. To do this we developed a protocol that uses geometric morphometric techniques to capture those aspects of bifacial point shape that might be behaviourally meaningful. As bifacial points have few landmarks, we developed automated ways to orient pieces in terms of homologous technological axes and then defined geometrically correspondent landmarks in accordance with these axes [60,62]). Our measurement protocol for bifacial points builds upon the three dimensional work of Lycett and colleagues [81–84] with some novel strategies that we believe suit our particular artefact collection, as well as assist in capturing the subtle aspects of shape that may be behaviourally interesting within this specific collection. Broadly speaking we oriented pieces in terms of their tip/base axis and then defined equidistant semilandmarks along each edge of each tool. We then used this set of points to project surface meshes of geometrically correspondent points on each surface (each “face”) of each biface adapting algorithms that have previously been applied successfully to study biological shape variation of bones and teeth (for reviews see [37,85]).

We describe the details of this process below.

### Data capture

Each bifacial point was scanned with a NextEngine surface scanner using NextEngine High Definition (“HD” 2011) data capture software. The bifacial points were mounted to rotate on their tip/base axis 16 times, for individual scans to be captured at 22.5 degree rotations. The non-artefact parts of the 3D model were then trimmed and the 16 individual scans were fused to produce a single three dimensional surface for each point. In addition to the surface scan, each point was photographed from the bilateral and the bifacial view as a base-line for subsequent cleaning of the models using a DSLR camera mounted on a copy stand with a 55 mm macro lens.

Unless otherwise stated all statistical tests, plots, and data management procedures described were produced in R [86]. Each 3D file was saved in PLY format and projected using functions available in the *Rvcg* package and the *Morpho* package(both packages: [87]) in order to view whether substantial holes existed on the scan surfaces. The Mesh-Doctor tools in Geomagic Studio Version 12 (1996–2010) were used to fix tunnels and non-manifold edges. The standard hole-filling interpolation allows for neighbouring surface convexities to be incorporated into the hole filling procedure (using the “Curvature” parameter). This function was used to fill any existing small holes on the surface. Filled areas were checked against photographs to insure fidelity to the original artefact. Hole filling was required on only a small number of specimens (N<10).

### Orientation

**Fig 3:**
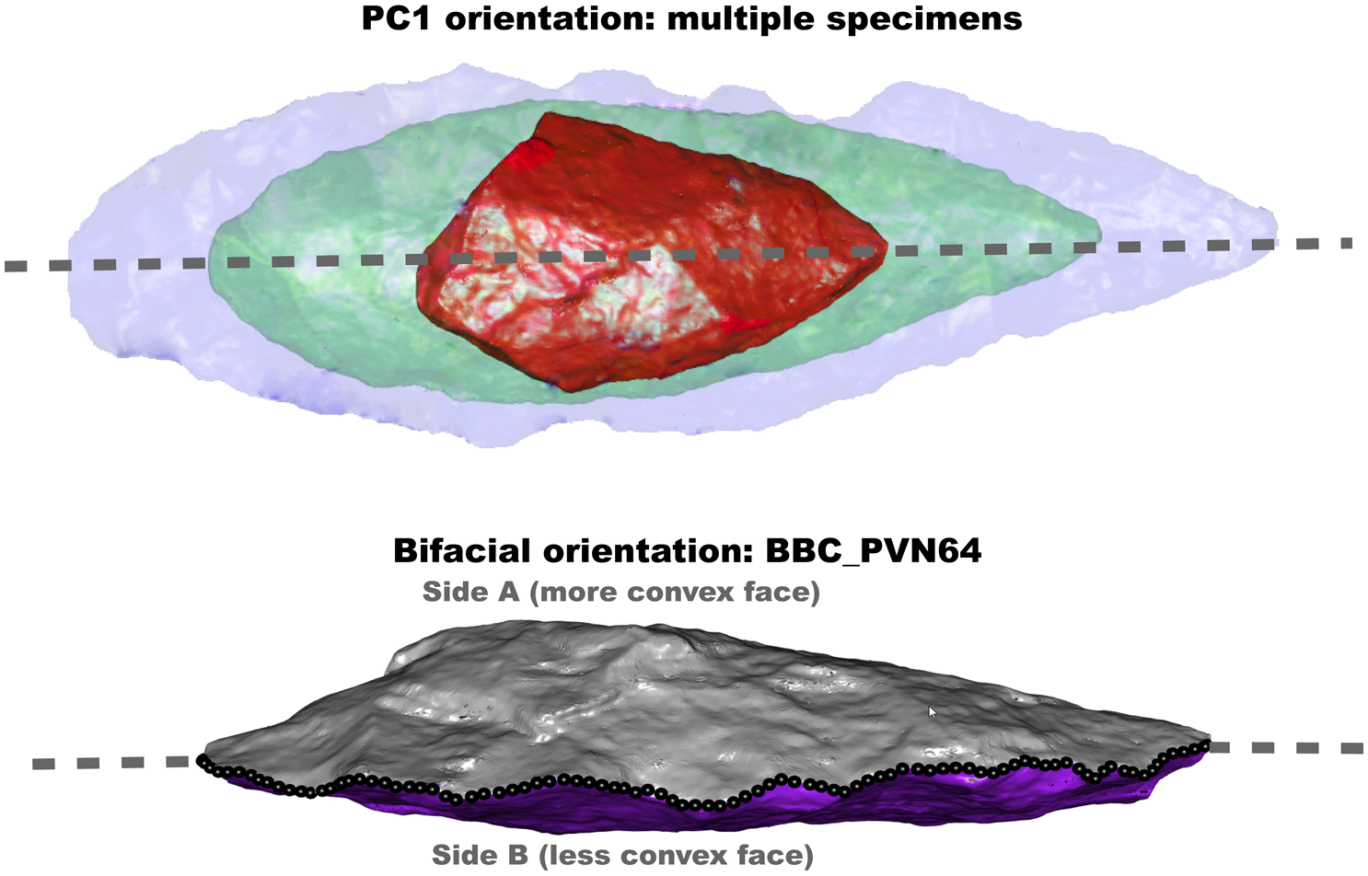
Demonstration of how points orient relative to one another along principal component one (after step 4.2(1)), as well as how the two faces of a biface are segmented along the edge (specimen BBC_PVN64).

The points were then oriented automatically with a newly developed R script. In a small number of specimens, the procedure described below did not provide a desirable orientation in one axis. In these cases the incorrectly oriented axis was manually inverted. The procedure is as follows:

1. A Principal Components Analysis (hereafter “PCA”) was conducted on the XYZ values of all the vertices of each individual scan. PCA essentially identifies the length axis of the biface as principal component one (hereafter “PC1”), which is the major axis of variation in the distribution of vertices for each scan. PC2 aligns with the width of the artefact, and PC3 aligns with the thickness of the artefact. Overall, the PCA places each bifacial point in the same orientation with reference to the main axes of variation in individual sets of vertices (Fig 3). This method for orientation has been shown to best replicate manual biface orienting techniques with the advantage that it is fully replicable [88]. However, as the orientations of PC axes are arbitrary, PC1 determines only the direction of the length axis and not which end is the tip.
2. We reoriented the specimen in accordance with the PC axes while maintaining the original triangulation of the surface vertices.
3. The bifaces could then be segmented along the long axis (PC1) into 100 sections so that the distance from one edge to the other could be calculated as the difference in the PC2 values within a specific segment.
4. Next, 50 parallel edge to edge distance measurements were calculated in the negative and in the positive spaces along PC1.
5. Two different checks were implemented to orient the tips of each biface in a consistent direction within PCA space. First, based on the assumption that the tip should be narrower than the base, the mean edge to edge distance measurement and the standard deviation in edge to edge distance were calculated for each half of the biface as defined in step 4. The pieces were then rotated 180 degrees if the tip was in the negative space along PC1.
6. A second check was made to ensure that the surface or biface ‘face’ with maximum convexity was consistently oriented in PCA space. Because discussions of plano-convex opposition in certain bifacial forms are exclusively qualitative in nature e.g. [53], it was necessary to experiment to determine which quantitative parameters best matched this qualitative distinction. In other words, it was necessary to consider which measure of “face size” best identified the face that one would identify as “the most convex face” just by looking at a specimen. For example, on Fig 3 it is clear that the silver face (“A”) is the most convex face: it was necessary to develop a measure that picked up on this. In the end, we defined two measures using the central axis of each specimen (Fig 3). In the lower specimen in Fig 3, the approximate position of the two faces (A and B) is illustrated relative to the central axis or central plane. Two stipulations were then tested: (a) that the face with the largest *mean* distance from the central axis was always facing in the same direction in PCA space, and (b) that the face with the *maximum* distance for a vertex point from the bifacial plane was similarly oriented. In the cases where (a) and (b) generated different results, the difference between the convexity of the two surfaces based on qualitative observation was negligible.
7. An important proviso is that in the study of an assemblage of highly variant forms where no automated process of orientation can be assumed to be uniformly successful, it was necessary to check manually how well the applied conditions were met. Biface vertices were plotted in PC’s 1 and 2 and in PC’s 1 and 3, printed to a PDF file, and then manually checked to see which conditions worked in righting faulty orientations on specific specimens.
8. The oriented vertices were then matched with the face matrix from the original scan so that a new PLY file of the oriented artefact could be written. All subsequent geometric morphometric operations were based on this file.

### Edge semilandmark determination

**Fig 4:**
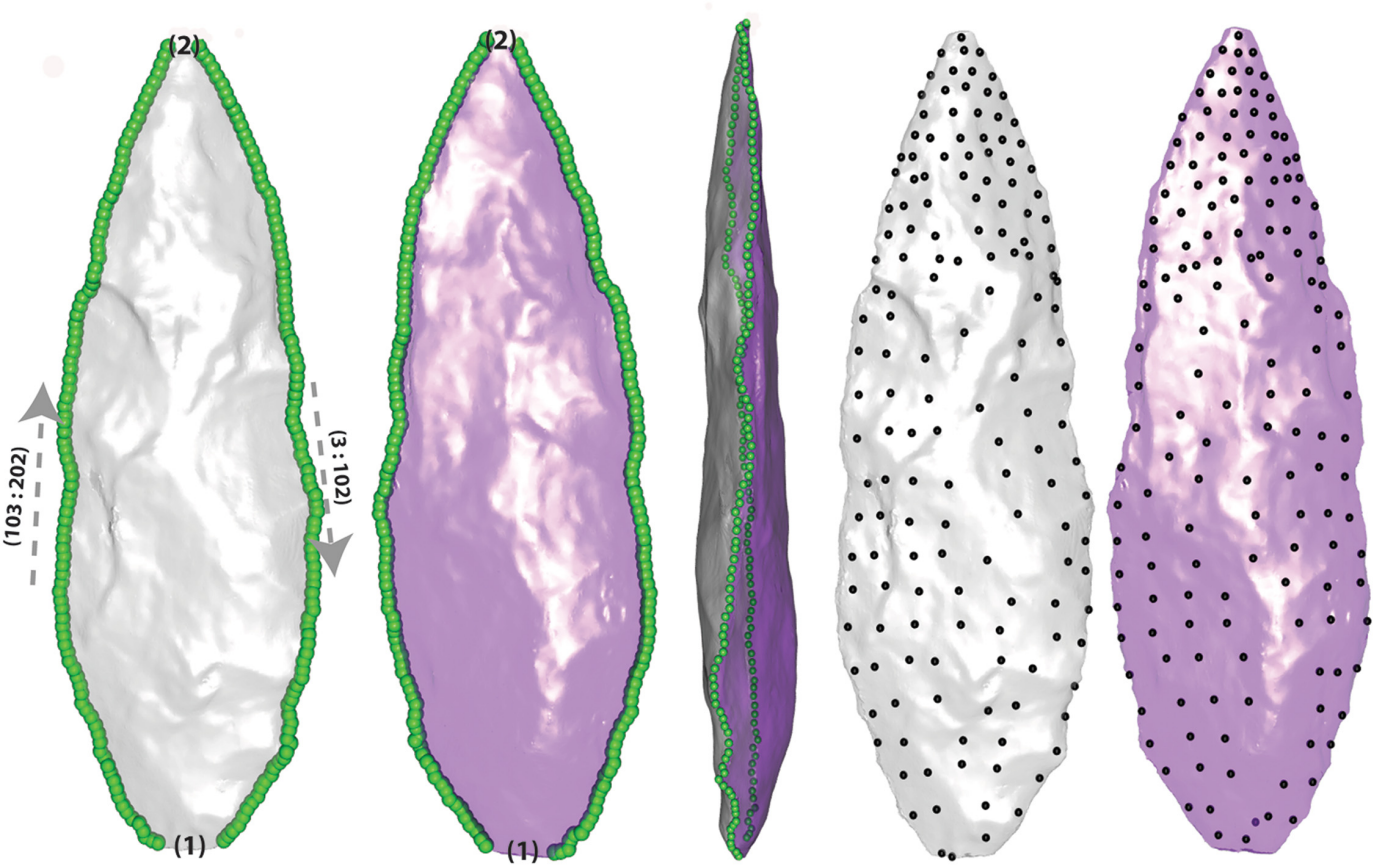
A point from Blombos (specimen BBC_P54) that illustrates the way in which the protocol segments the piece in accordance with two faces (different colours here distinguish the faces), marks the tip and base (landmarks (1) and (2)), maps semilandmarks on the edge (green landmarks) and surface points on each of the faces (black landmarks). This particular point comes from layer “cc” in the M1 Lower chronological sub-phase.

All geometric morphometric analyses require the same number of measurement points in corresponding locations on every specimen. We therefore used the following protocol to place landmarks and semilandmarks along the edge, as well as surface semilandmarks on both faces of all artefacts. The tip and base of each biface, defined as the extreme positive and negative values on PC1, were documented as landmarks 1 and 2 (Fig 4). The vertices on the edge of each piece were then determined by following the steps described in (5) and (6) above. As these edge vertices are unordered, we employed a travelling salesman algorithm (using the function FindShortestTour in the software Mathematica 10, Wolfram Research) to determine a continuous sequence of edge vertices. We then reordered this sequence so that the curve along the edge would start at the artefact’s tip (i.e. landmark 2) and run clockwise via the landmark on the base all the way back to the tip. The edge semilandmarks were subsequently resampled in order to evenly space these points. This was done following the algorithm described in Gunz et al. [89] and Gunz and Mitteroecker [85]. First, we computed a cubic spline function through the reordered sequence of edge vertices, and then used a dense set of points obtained from this cubic spline function to measure the lengths of the curve segments between the landmarks on the tip and base (landmark 2 and landmark 1, respectively) on either side. Finally, we placed 100 equidistant curve semilandmarks (hereafter “edge semilandmarks”) on either side of the artefact (Fig 4). This procedure resulted in 202 correspondent 3D landmark and semilandmark coordinates on each biface.

### Semilandmarks on surfaces

Next we placed a mesh of surface semilandmarks on both faces of each artefact. To this end we first separated the two faces of each artefact along the bifacial edge based on the surface normals computed for each triangle. Figs 3 and 4 illustrate how this step segments a biface along its edge. Each face was then written as a separate differently coloured PLY file (Fig 4). The two faces for each specimen were then cleaned manually with Geomagic Studio 2012’s Meshdoctor tools, and automatically cleaned with functions from the R *Rvcg* package [87]. After these steps, the two separately coloured halves were united to see whether the region where the colours met accurately approximated the bifacial edge of each piece. Within this phase a small number of problems arose that were associated with step and hinge terminations on the bifaces. These resulted in the distal part of a small number of flake scars having surface normals oriented in directions mistaken for the opposing face of the specimen in the procedure outlined above. In these situations interpolation of the tiny holes left by distally stepped areas was sufficient to approximate the original specimen surface, which was available for comparison.

On a randomly chosen biface we manually digitized a mesh of 154 evenly spaced 3D coordinates on the front face using the software Avizo (Visualization Sciences Group). Using a thin-plate spline interpolation function computed from the 2 landmarks and the 200 edge semilandmarks described above, we warped this template mesh to all artefacts of the collection. These warped points were then projected onto the back face, by selecting the closest vertex from the respective PLY file; these 154 projected points were treated as surface semilandmarks. The form of each artefact is thus described by 510 geometrically corresponding three-dimensional coordinates: 2 landmarks on the base and tip, respectively; 200 edge semilandmarks, and 308 surface semilandmarks.

### Multivariate analyses

The three-dimensional coordinates were subjected to Procrustes superimposition [90], which standardized scale, orientation and location. The resulting shape variables were analysed using principal component analysis on the assemblage of individual landmark configurations (PCA), and multiple regression analysis. In addition to standard PCA, *between group* PCA analyses [91], were also used to draw out and visualize differences between the groups of bifacial point forms associated with each of the stratigraphic sub-phases at Blombos (hereafter “Group PCA”). Group PCA calculates a covariance matrix based on group means—here the mean bifacial point shape within each of the stratigraphic sub-phases—and then re-projects the original observations into the eigenspace of this new covariance matrix.

The effect of the independent variables of stratigraphic layer and size on PC1 shape was estimated using a multiple regression. Additionally, permutation testing of differences in mean point shape along PC1, specifically, between groups of points within the different chronological sub-phases and layers was conducted. The permutation tests compared the distances between the mean shapes associated with each sub-phase relative to the random assignment of cases to each sub-phase (using 10000 iterations).

Further, we predicted that the nature of the relationship between size and shape change potentially varied systematically through time. Stratigraphic layer, centroid size and their interaction were, therefore, included as test predictors within the model. To control for the effects of variation in raw-material, raw-material type was also included as a control predictor in the model.

As the frequency of different raw-materials varied substantially through time (Table 1), it is possible that raw-material type influenced potential patterns of shape variance through time. If raw-material was driving the pattern of variation through time, one would expect the interaction between raw-material and stratigraphic level to be significant within the multiple regression, i.e. one would expect that raw-material type influenced the effect of level on PC1 shape. Another model was therefore formulated to look at the possible influence of raw-material on point shape change through time.

Before fitting the models, the distributions of the covariate centroid size and the response were checked. Centroid size was subsequently log-transformed to achieve a more symmetrical distribution. All covariate predictors were then z-transformed to a mean of zero and a standard deviation of one to have comparable estimates and a more easily interpretable model with regard to the interactions [92]. A Qqplot and scatterplot of the residuals against the fitted values were visually inspected to check the assumptions of the model, which are normally distributed residuals with homogeneous variance. No deviations were suggested by the plots. Model stability indicators such as leverage, dffits, dfbetas and Cook’s distance also did not suggest the existence of substantially influential cases. Variance Inflation Factors indicated that colinearity was not an issue (largest VIF = 1.101, [93–96]).

As an overall test for the significance of the interaction between size and stratigraphic layer the fit of the full model was compared with that of the reduced model which lacked the interaction between size and stratigraphic layer but included all other terms. This comparison was based on an F-test (R-function ANOVA with the argument test set to “F”). The model was fitted in R using the function “lm” and VIF was determined using the function “vif” from the package “car” [97].

### Expectations

Broadly speaking raw-material variability can have an influence on the range of lithic forms we see in the archaeological record [98–103]. If raw-materials play a role in structuring variability within bifacial points, we expect clustering of point shapes by raw-material type on the PCA plots. We can also test the precise effects of raw-material on bifacial point form more directly by formulating models using raw-material type as one of the predictors and bifacial point form (here shape change on PC1) as a response.

Time or the contemporaneity of specimens within the stratigraphic sequence could also influence shape if particular shapes were produced systematically at different times within the Still bay at Blombos. Again, we expect that if bifacial form varies with stratigraphic position, these points will cluster together on the PCA.

If form does change through time, one possible expectation is that through time points will become more *standardized*. Lyman et al.[104] summarises a set of expectations including that (1) artefact class persistence through time tracks heritability, (2) noticeable variation is introduced by copying error and experimentation [105,106], and that (3) selection reduces or stabilises variation [107,108]. Through time—holding population size, ecology and reduction intensity constant—one would expect variation in bifacial point form associated with innovation and experimentation to decrease, either through drift or selection [104,109]. A more specific expectation is that we would expect an increase in standardization through time to be reflected by a decrease in the coefficient of variation of PCA scores for ranges of points in younger layers, relative to older layers. Yet it is possible that *selection* and *reduction intensity* have similar effects in terms of how they influence variation in shape within a layer. If modal point form is more reduced in certain layers one might expect variation to be lower regardless of the possibility that selection was reducing variation.

Finally, we do not expect that these potential drivers all have ‘main effects’ on modal bifacial point form. We expect that the effect of one influential variable—such as raw-material type for example—has an influence on another predictor such as blank type, and that blank type in turn has an influence on the range of bifacial point forms produced. There may be significant *interactions* between individual drivers of bifacial point shape, and these interactions can only be tested for by formulating models for this purpose.

## Results

The variance table from the PCA showed that by far the largest amount of variation in the dataset was captured by PC1 (Table 2). The subsequent components each account for substantially less variance (< = 9%), and overall each accounts for a very similar proportion of variance in the dataset. This suggests that the structure of variance within the components subsequent to PC1 is not very stable, i.e. the removal of small numbers of cases from the population may result in a re-ordering of these subsequent components. For this reason, only morphological variance associated with PC1 will be interpreted here. We plot PC2 below for demonstration purposes.

**Table 2:** The first 6 principal components.

Components	Eigenvalues	% Variance	Cumulative%
1	0.005	33.685	33.685
2	0.001	9.001	42.685
3	0.001	7.260	49.946
4	0.001	5.201	55.147
5	0.001	4.495	59.641
6	0.001	4.212	63.854

**Fig 5:**
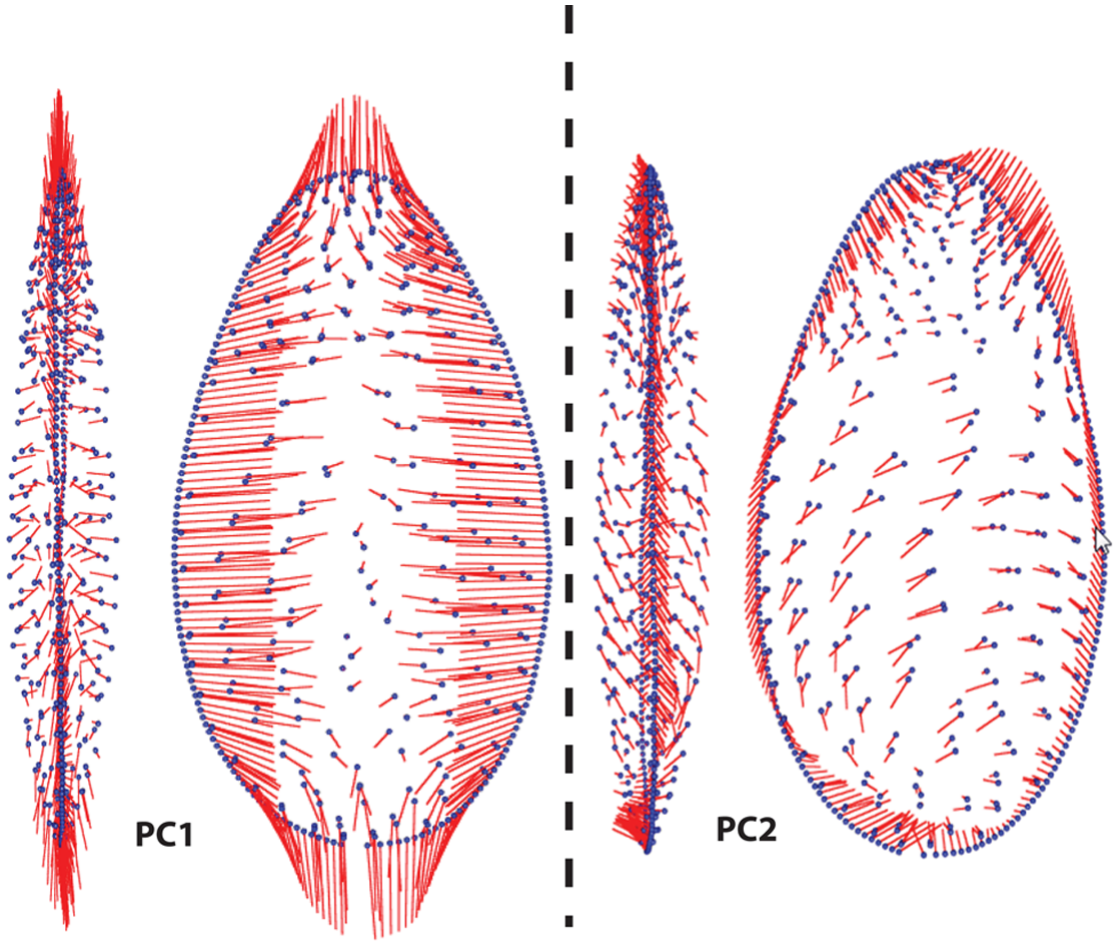
Vectors showing the landmark configuration deviation between the mean shape in the collection (the blue outlines) and the negative space on both PC1 and PC2. The plot was devised using the function ‘pcaplot3D’ in the Morpho package [87].

Fig 5 visualises the major axes of variation in artefact form as computed by the PCA. What this model shows is vectors plotting the extreme negative shape onto the mean shape in the collection. Shape variation captured on PC1 comprises a continuum between two forms. In the extreme negative space are bilaterally elongated foliate and lanceolate forms that retain similar outlines in bifacial and bilateral profile. The maximum thickness is located at a distance of approximately 1/3 of the length axis from the base in these elongated forms. The extreme positive end represents a range of ovate forms where the maximum thickness in bifacial profile is located at approximately 1/2 of the length axis from the base. Thus PC1 represents the combination of bilateral elongation and bifacial refinement. When points tend to be more elongated in their tip and base regions, they also tend to be more bifacially refined within their mid-sections. These three dimensional aspects of shape change will hereafter be referred to as “shape change along PC1” or “elongation in combination with refinement”.

PC2 seems to be driven by the tendency for bifaces to have “s-shaped” edges in combination with slightly off centred or asymmetric tips in the negative space and more bilaterally symmetrical forms with straight edges in the extreme positive space (Fig 5). The behavioural drivers behind the tendency for bifaces to have “s-shaped” edges have been a subject of debate in handaxe studies [71]. However, we do not further explore this aspect of biface morphology here as it accounts for a relatively low proportion of variance in the dataset (9%).

**Fig 6:**
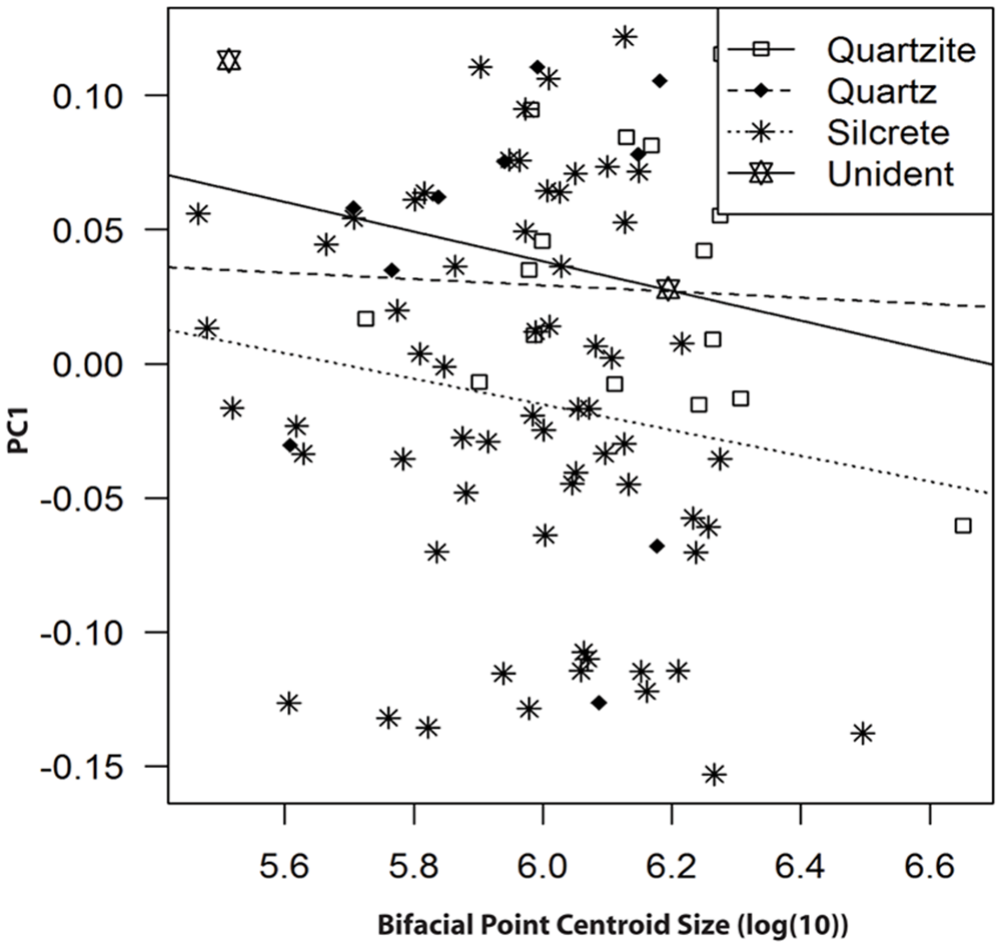
PC1 plotted against bifacial point centroid size for each of the raw-material groups in the entire collection. The subtle slope of the regression indicates that the effect that does exist may possibly have to do with the reduction of silcrete and quartzite points from large and elongated to small and ovate.

Fig 6 shows a regression of PC1 against bifacial point size for each of the raw-material groups in the collection, and indicates that there is very little influence of size on PC1 when considering the entire collection. Tests for a correlation between centroid size and PC1 scores were not significant. That is, bifacial point size does not represent a significant effect on shape as quantified by PC1 (Spearman’s = -0.06, S = 66458, P = 0.56). The subtle slope that does exist may have to do with the resharpening of silcrete and quartzite points which turns large and elongated forms into small and ovate forms, however, other measures of bifacial reduction within the collection need to be considered in order to clarify this inference.

**Fig 7:**
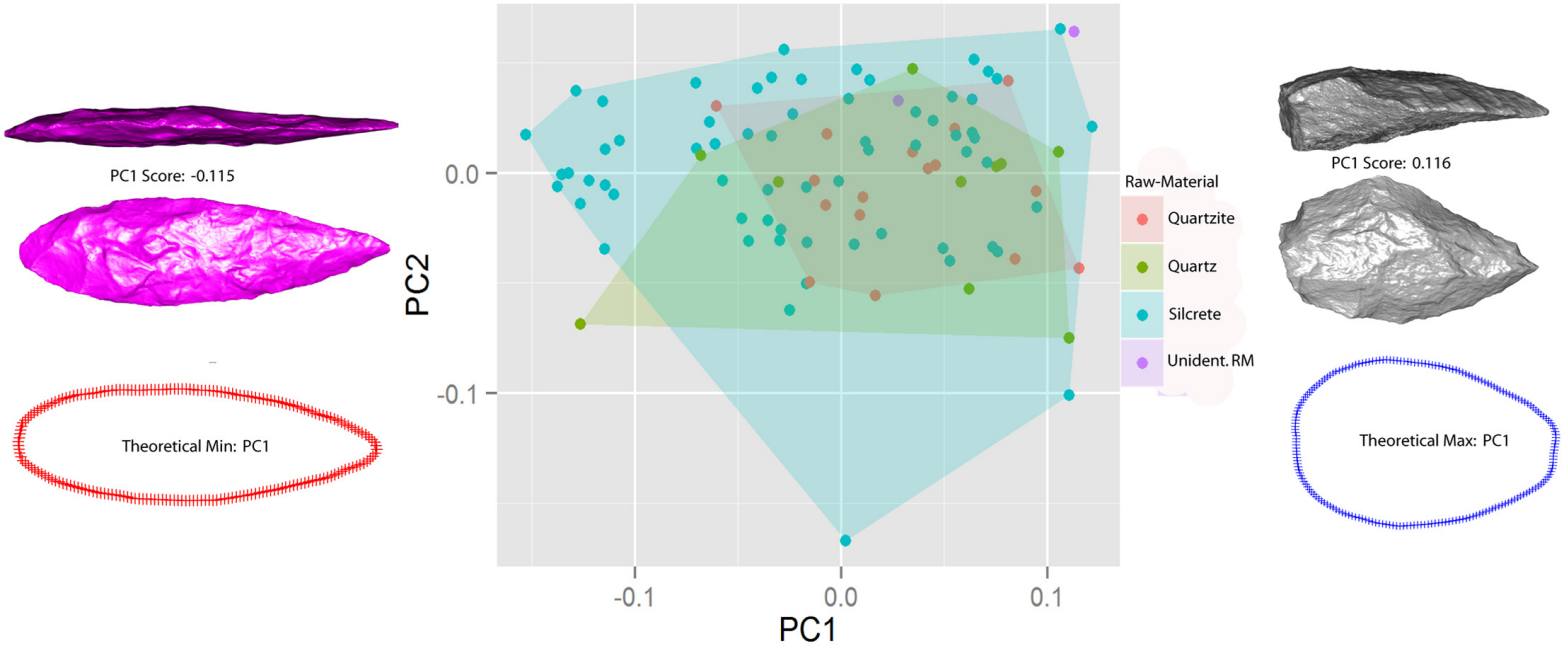
PCA of Still Bay bifacial points showing the first two axes of variation with raw-material type as a grouping factor. Convex Hulls of each group show that points made on different raw-materials overlap on the major axes of variation. Artefacts are plotted at the extremes of PC1 for illustration purposes. Additionally the red and blue outlines indicate the two-dimensional edge configurations of computed theoretical shape extremes on PC1. Point scan models are not to scale.

Results of the PCA were visualized using a bivariate plot of factor scores for components 1 and 2, using raw-material as a grouping factor (Fig 7). Convex hulls were plotted for each raw-material group, and they represent the variation in form for bifacial points made on different raw-material types. The convex hulls indicate that there is both substantial overlap and relative separation between the ranges of shapes in different raw-material groups along PC1 and PC2.

**Fig 8:**
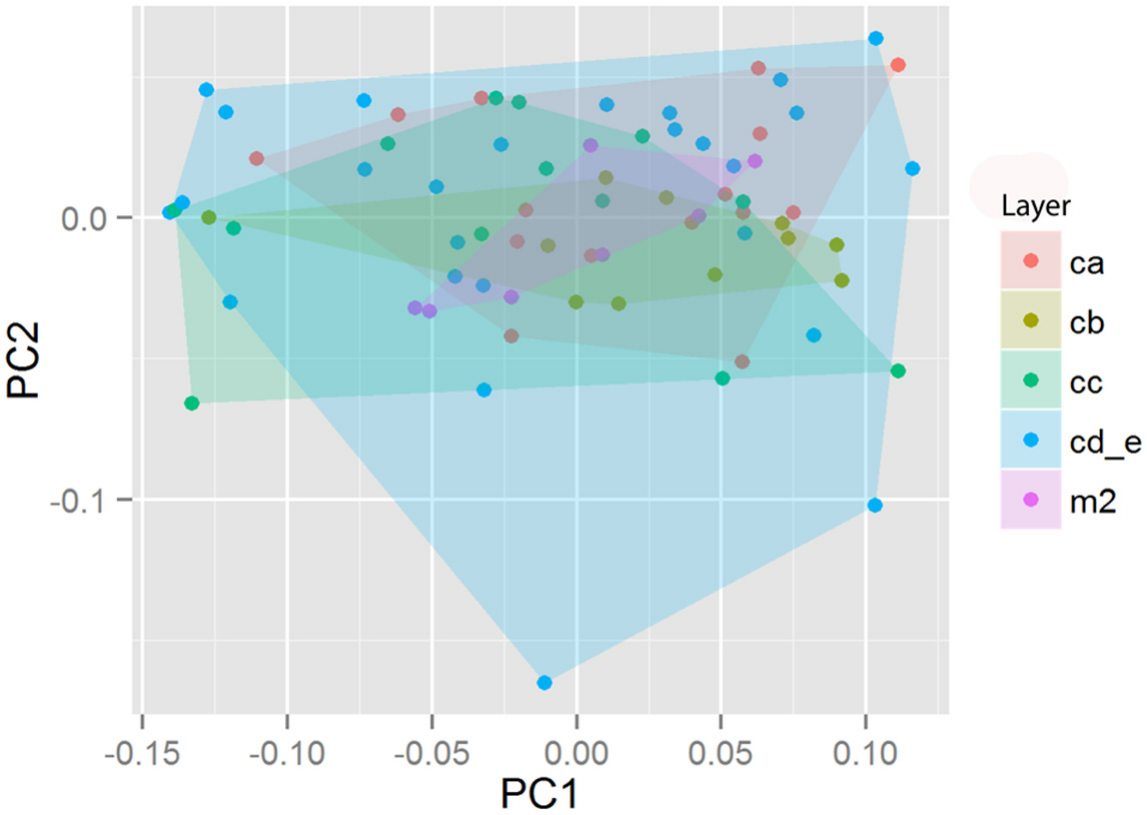
PCA of Still Bay bifacial points showing the first two axes of variation with stratigraphic layer as a grouping factor. Points within different units overlap but seem to have different shaped distributions along PC1.

A bivariate plot of PC1 and PC2 scores was also made using stratigraphic layer as a grouping factor, represented by convex hulls (Fig 8). The stratigraphic layers are arranged in the key from youngest to oldest. “Layers” here are distinguished from chronological “phases”, each of which comprises multiple layers (see above section on “Layer splitting structure”). There is also substantial overlap between the ranges of point shapes in different stratigraphic layers.

**Fig 9:**
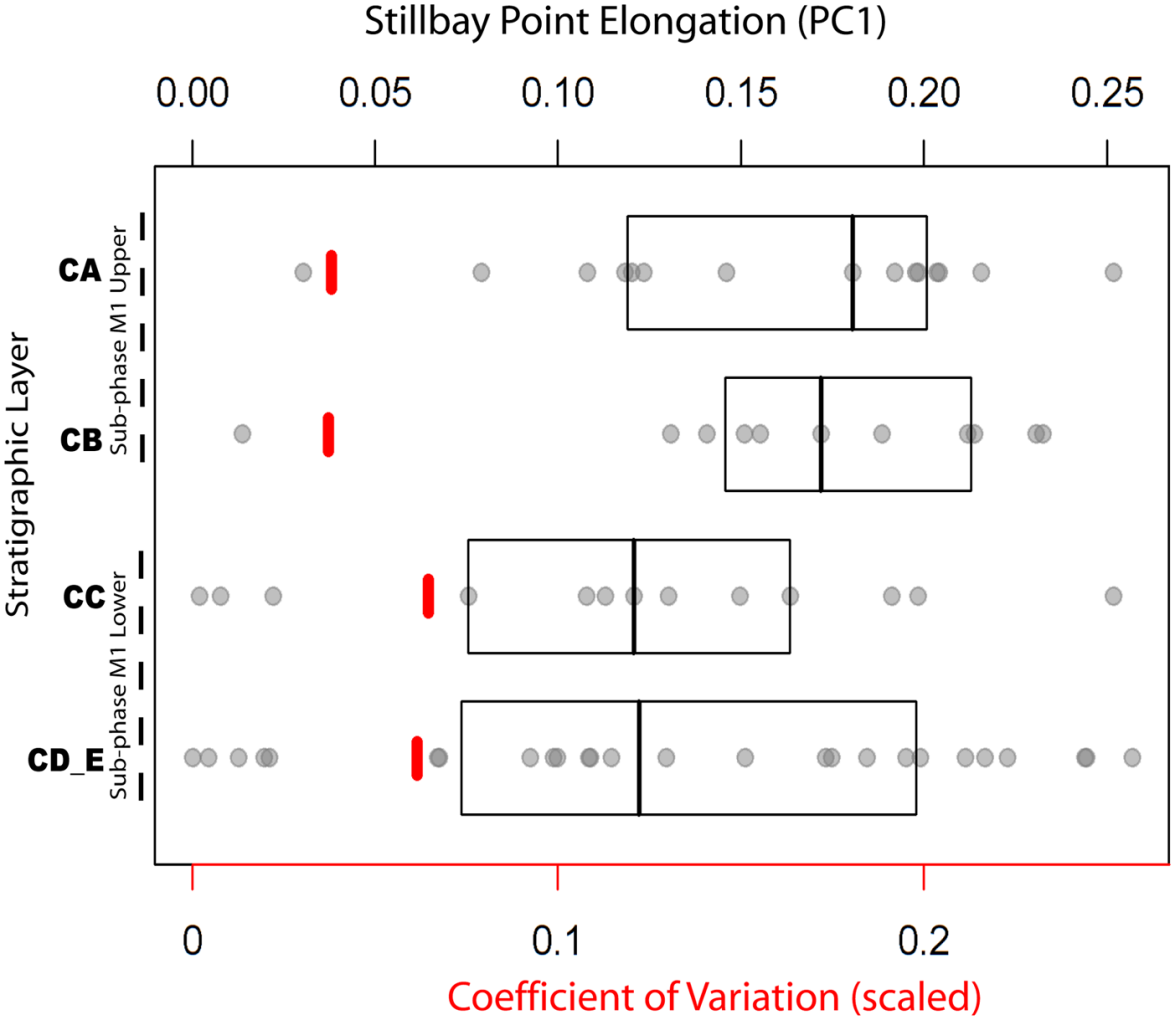
Principal component scores for points within each layer in the BBC M1 phase. There are differences between the points in the M1 Lower (layers cd_e and cc) and M1 Upper (layers ca and cb) sub-phases. The M1 Upper points are more elongated and more standardized in their overall morphology (here plotted as scaled coefficient of variation) than the M1 Lower points. The central lines in the rectangles refer to medians whereas the rectangles themselves mark out the quantiles of the ranges of scores per layer.

The points in the lower M1 phase are substantially less standardised and have different scores along PC1 relative to the points from the upper layers (Fig 9). A plot showing PC1 scores by layer and associated coefficients of variation is used here to visualise differences in the way variation in shape is structured through the upper part of the sequence. The ranges of points within layers CA and CB (representing the M1 Upper phase) have more elongated forms and, more importantly, lower coefficients of variation which indicated that they are more standardized in form than the points in the M1 Lower phase layers. Permutation testing indicated significant differences in mean point shape along PC1 between the M1 Upper sub-phase and the M1 Lower sub-phase groups of points (n = 72, dist = 0.034, p = 0.048). However, comparisons of the M2 sub-phase with either the M1 Upper or the M1 Lower sub-phases were not significant (M2: Upper M1, n = 72, dist = 0.022, p = 0.475) (M2: Lower M1, n = 72, dist = 0.012, p = 0.690).

**Fig 10:**
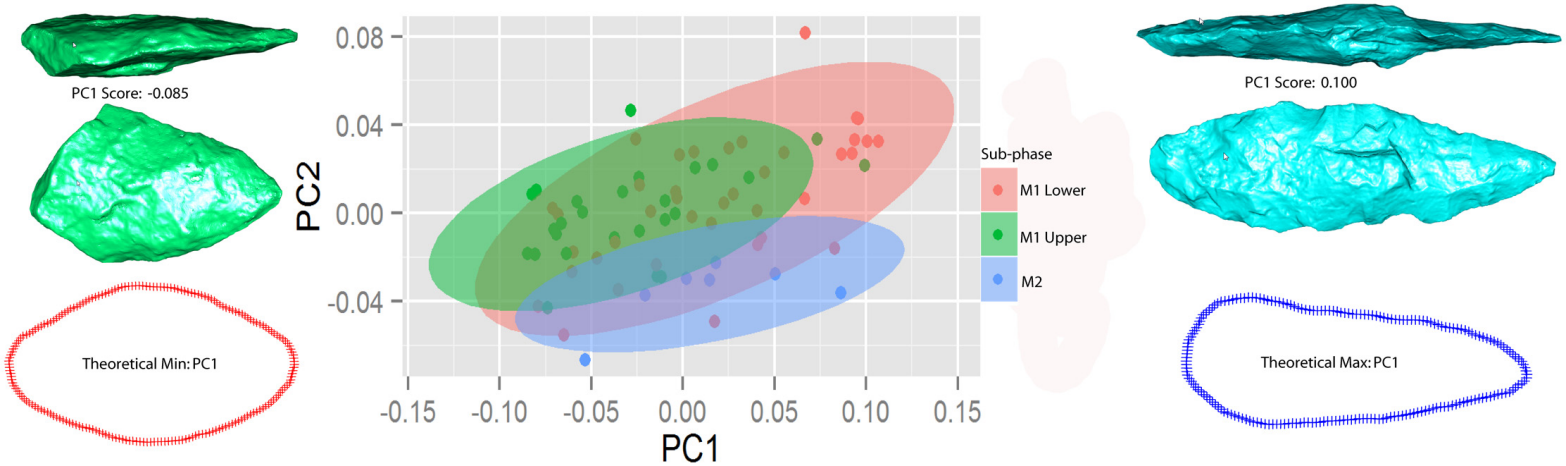
Group Principal Components Analyses using the two M1 sub-phases—M1 Upper and M1 Lower—as grouping factors. The small sample of points (7) from the M2 phase is also included. Polygons indicate 95% confidence intervals for each group. The bifacial points at the extremes of PC1 illustrate the two extremes of shape within this plot. The fact that the major axis switches—in terms of which end represents more elongate forms—relative to the standard PCA plot is of no consequence. The artefact in the extreme negative represents a specimen that appears to have broken during an advanced stage of manufacture or maintenance and then recycled post-breakage. The red and blue outlines indicate the two-dimensional edge configurations of computed theoretical shape extremes on PC1, on this Group PCA. Point scan models are not to scale.

A Group PCA was then conducted using stratigraphic sub-phase as the grouping factor (Fig 10). The M1 Upper phase includes layers CA and CB and the M1 Lower phase includes layers CC, CD and CE. In addition, the small collection of points from the M2 phase was used as a third control group in the plot. However, we do not interpret the distribution of the M2 group plot here because of the small sample size.

The multiple regression investigating the interaction between raw-material and stratigraphic layer indicated no significant interactions (see Table 3 of coefficients for this model).

**Table 3:** The estimates and associated t and p-values for the model investigating the interaction between raw-material and stratigraphic layer.

	Estimate	t value	p
*Intercept*	0.038074	1.776	0.0806
*Size*	-0.008755	-0.923	0.3595
*Level*	-0.008813	-0.483	0.631
*Quartz*	-0.022013	-0.616	0.5404
*Silcrete*	-0.052054	-2.176	0.0333
*Raw-mat Unident*.	-0.007434	-0.103	0.9183
*Level*:*Quartz*	0.027704	0.854	0.3965
*Level*:*Silcrete*	0.015958	0.75	0.4561
*Level*:*Raw-mat RMUnidentified*	0.050317	0.705	0.4834

**Fig 11:**
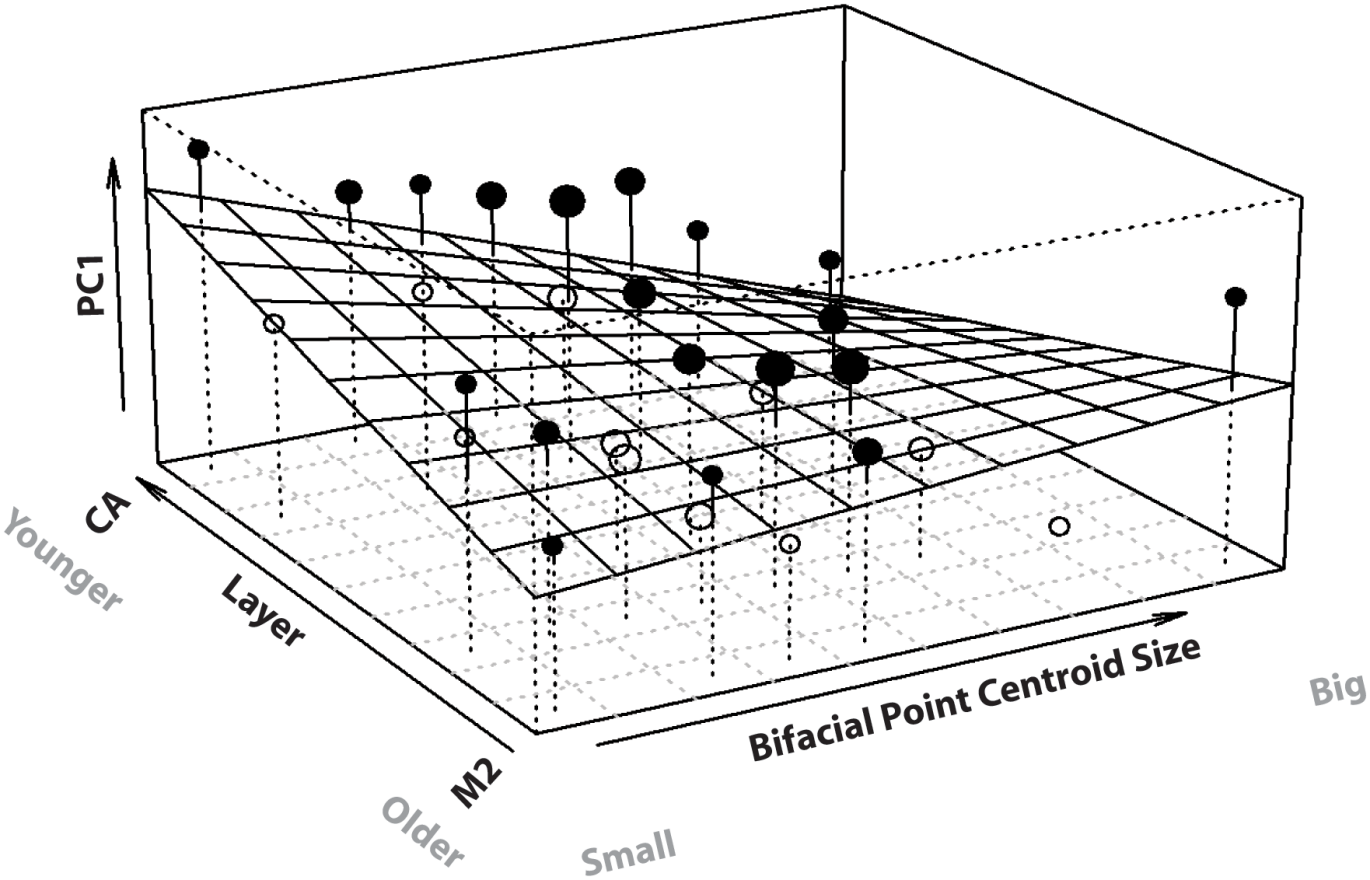
Plot showing the interaction between bifacial point stratigraphic layer and bifacial point centroid size, as well as their combined effect on PC1 shape change. Importantly, the “stratigraphic layer” variable has been specifically transformed for usage in the plot: the individual cells along this axis cannot be equated with individual layers, however, the axis represents the spectrum of variation in the stratigraphic layer variable. In the older layers there is little to no relationship between bifacial point size and shape change along PC1. However, in the younger layers point elongation and refinement are correlated with overall point size. Data points on the plot reflect the average response per cell and are scaled according to their size in accordance with the number of data per cell. Data points are depicted as filled when their average is above the fitted model and empty if they fall below.

In the multiple regression investigating the interaction between size and stratigraphic layer, the full-reduced model comparison indicated that the interaction was very close to being significant (F = 3.578, P = 0.06) (Table 4). For many statisticians, a “marginally non-significant p-value” indicates some evidence against the null-hypothesis [93,110]. Therefore, size had some influence on the tendency for points to be elongated and refined within the Blombos collection. However, the effect of decreases in point size on decreases in elongation and refinement was not the same for all stratigraphic layers (interaction: estimate+SE = -0.01+-0.005, t = -1.89, P = 0.06). This suggests that the way the interaction operates should be investigated through plotting the relationship between the variables of shape, size and stratigraphic level (Fig 11).

**Table 4:** The estimates and associated t and p-values for the model investigating the interaction between stratigraphic layer and size.

	Estimate	t value	p
*Intercept*	0.031	*	*
*Size*	0.019	*	*
*Level*	0.006	*	*
*Quartz*	0.033	-0.954	0.344
*Silcrete*	0.024	-2.597	0.012
*RMunidentified*	0.056	-0.304	0.762
*Size*:*Level*	0.006	-1.892	0.063

“*” Values are not included as they do not have a meaningful interpretation due to being included in the interaction.

## Discussion and Conclusion

### Analyses interpretation

The convex hull polygons that represent raw-material groups indicate that bifacial points produced within each raw-material type have forms that substantially overlap with one another on the major axes of variation in the PCA (Fig 7). However the linear model does indicate that whether or not a bifacial point was manufactured on silcrete influenced its position on the major axis of shape variation. In other words, the PCA indicates that points made on different raw-materials have shapes that substantially overlap with one another on the PCA. However, this does not imply that the ranges of shapes within different raw-material groups have similar distributions on the PCA. Although shapes overlap between raw-materials, there are also clearly differences with regard to how shapes within raw-material groups are distributed, and the linear model suggests that these differences are significant.

The standard PCA indicates that there is substantial overlap between the points from different layers, but it also suggests that points from different layers have different distributions of variance on the PCA. Further, on the between-group PCA using stratigraphic sub-phase as the grouping factor, 95% ellipses show that different sub-phases have different mean point shapes. In addition, the range of shapes in the M1 Lower sub-phase is substantially more variable than the range in the M1 Upper sub-phase (Fig 9).

The plot depicting the multivariate regression model indicates that stratigraphic layer has an influence on the effect of size and, in turn, on shape change along PC1 (Fig 11). However, the effect of size on shape is substantially more pronounced in the upper point bearing layers—CA and CB—even though the older points are more variable overall. In the lower layers (CD_E and CC), size seems to have relatively less influence on variation along PC1.

The variation in the upper levels reflects the reduction in size of points from larger and elongated to smaller and, potentially, more extensively reduced ovate forms. However, interpreting patterns of biface shape and size change in this way entails assuming that points started out being broadly similar or comparable sizes.

An untested hypothesis is that variation between elongated and ovate forms in the lower layers may be associated with varying stages of manufacture, and possibly even design imperatives associated with manufacture. Modal variation in the lower layers looks to be between larger ovate like roughouts and smaller elongated forms. In the lower levels variation among points may be structured to some extent by the initial phases of bifacial point manufacture and the discard of points during the manufacture process (a possibility suggested by Henshilwood *et al*.[2] who observed points broken during manufacture in the lower layers but not in the upper layers).

Interestingly, silcrete is significant in the model (estimate+SE = -0.061+-0.02, t = -2.59, P = 0.01). The drop in PC1 scores between the M1 Lower sub-phase and the M1 Upper is associated with a drop in silcrete proportions in the bifacial point collection from 79.4% (M1 Lower) to 50% (M1 Upper).

### Interpreting variation in shape

Here we investigated the influence of raw-material variability and tool size on bifacial point form in a way that factored in variations in how these variables may have responded to changes in the ways hunter-gatherers interacted with their environments through time. We attempted to compartmentalise the components of shape variability that raw-material availability and tool size—potentially reflecting degree of tool reduction—seem to explain.

Analyses of changes in point shape and size through time revealed a complex pattern. To recap, several trends in variation in bifacial point morphology regarding the transition from the M1 Lower (M1b) to M1 Upper (M1a) sub-phases at Blombos were documented. These include an increase in standardisation in bifacial point morphology as well as a systematic change in elongation and refinement through time.

The expectation that size has a linear effect on elongation and that decreases in both reflect greater intensities of reduction does not fully explain the identified pattern. In the lower layers larger ovate like bifacial forms grade into smaller, more elongate and refined bifacial points. The visa versa relationship between size and shape exists in the upper layers.

We cannot exclude the possibility that the rise in point shape standardisation through the sequence may be related in some respect to raw-material variability. Overall variation in point shape may possibly correlate to (1) variation in the usage of silcrete to manufacture points as well as potentially (2) how intensively points on all raw-material types were being manufactured and reduced on site at different time-periods represented within the sequence. However, inferences about (2) require complementary avenues of evidence concerning how characteristics of point morphology relate to bifacial point size and different phases of point life history.

If one accepts the chronological shift in standardization to be the product of cultural transmission, we may expect this increasing standardisation to represent the “winnowing of less-efficient forms”([104]:4). However, increases in standardization may also be influenced by modal increases in point reduction, i.e. as points are generally more extensively reduced overall variability in point form may also be reduced, but this remains an untested expectation.

Here we developed a new analytical protocol to show that in the context of a single Still Bay locality, point morphology varies significantly through time. We documented systematic changes in bifacial point morphology that may be associated with ways in which—and the extent to which—finished points were maintained, through time. Interestingly, evidence for systematic change in site use through time within the Still Bay layers at Blombos is represented in other non-lithic components of material culture [31]. However, it is not clear how change through time reflected in different spheres of material culture in the Still Bay at Blombos relate to one another.

Clarifying the nature of these technological changes and testing further hypotheses about their behavioural drivers may require more detailed data regarding the specificities of how bifacial points were manufactured and maintained, the dimensional and morphological correlates of these practices, and how these variables interact with the geometric morphometric data presented here as well as other spheres of material culture represented within the Still Bay layers at Blombos. These objectives comprise challenging future avenues of this work on Still Bay bifacial point variability.

## Supporting Information

S1 File ID numbers of specimens analyzed within this study (Table 1). Principal component scores as well as raw-material and level affiliations used for plots in the manuscript (Table 2).
(DOCX)
