# Supplementary material for: Diachronic Change within the Still Bay at Blombos Cave, South Africa
Source: PLoS One. 2015 Jul 2;10(7):e0132428. doi: 10.1371/journal.pone.0132428 (PMC4489860; doi:10.1371/journal.pone.0132428)
Supplement: S1 File — Principal component scores as well as raw-material and level affiliations used for plots in the manuscript (Table 2). (DOCX) [file pone.0132428.s001.docx]

Supporting Information

The 119 specimens analyzed are housed within the, permanently accessible, Archaeology Division repository at the Iziko public Museum in Cape Town, South Africa. Permissions were sought by one of the authors (WA,) from the museum curatorial administration (Dr. Sven Ouzman), to study the collection at the museum although he was not involved in the excavation and recovery of the collection. The holding institution has a copy of the scans and interested readers can apply to the museum to access these scans or the actual artefact collection.

The artifact IDs follow specimen numbers allocated within the Villa et al. (2009) [1] initial analysis of the collection where these were available (the “P”, “PV” and “PVN” sequences), which seemingly represents two phases of analysis. The points labeled as “P” were initially assigned by Marie Soressi and those labeled as “P” or “PVN” represent Paola Villa’s numbering system [1: 444]. The specimens not labeled within this system – but which were nevertheless analyzed for this study - were numbered in accordance with stratigraphic information documented on the artifact specimen labels.

Table 1: Specimens analyzed within this study.

| 1 | BBC_G5A |
| --- | --- |
| 2 | BBC_ H5b L2_001 |
| 3 | BBC_ H5b L2_002 |
| 4 | BBC_MH15 |
| 5 | BBC_MH18 |
| 6 | BBC_MH19 |
| 7 | BBC_MH20 |
| 8 | BBC_MH22 |
| 9 | BBC_MH23 |
| 10 | BBC_MH24 |
| 11 | BBC_MH26 |
| 12 | BBC_MH27 |
| 13 | BBC_MH29 |
| 14 | BBC_MH30 |
| 15 | BBC_MH31 |
| 16 | BBC_MH32 |
| 17 | BBC_MH35 |
| 18 | BBC_MH39 |
| 19 | BBC_MH40 |
| 20 | BBC_P13 |
| 21 | BBC_P15 |
| 22 | BBC_P16 |
| 23 | BBC_P17 |
| 24 | BBC_P1 |
| 25 | BBC_P25 |
| 26 | BBC_P26 |
| 27 | BBC_P27 |
| 28 | BBC_P29 |
| 29 | BBC_P2 |
| 30 | BBC_P31 |
| 31 | BBC_P40 |
| 32 | BBC_P41 |
| 33 | BBC_P44 |
| 34 | BBC_P48 |
| 35 | BBC_P49 |
| 36 | BBC_P50 |
| 37 | BBC_P52 |
| 38 | BBC_P55 |
| 39 | BBC_P56 |
| 40 | BBC_P58 |
| 41 | BBC_P59 |
| 42 | BBC_P63 |
| 43 | BBC_P64 |
| 44 | BBC_P67 |
| 45 | BBC_P70 |
| 46 | BBC_P71 |
| 47 | BBC_P72 |
| 48 | BBC_P75 |
| 49 | BBC_p76 |
| 50 | BBC_P84 |
| 51 | BBC_PV84 |
| 52 | BBC_PV97 |
| 53 | BBC_PVN105 |
| 54 | BBC_PVN10 |
| 55 | BBC_PVN115 |
| 56 | BBC_ PVN 127 |
| 57 | BBC_PVN130 |
| 58 | BBC_PVN131 |
| 59 | BBC_PVN132 |
| 60 | BBC_PVN139 |
| 61 | BBC_PVN140 |
| 62 | BBC_PVN144 |
| 63 | BBC_PVN147 |
| 64 | BBC_PVN151 |
| 65 | BBC_PVN152 |
| 66 | BBC_PVN154 |
| 67 | BBC_PVN155 |
| 68 | BBC_PVN156 |
| 69 | BBC_PVN158 |
| 70 | BBC_PVN162 |
| 71 | BBC_PVN164 |
| 72 | BBC_PVN168 |
| 73 | BBC_PVN177 |
| 74 | BBC_PVN182 |
| 75 | BBC_PVN184 |
| 76 | BBC_PVN195 |
| 77 | BBC_PVN206 |
| 78 | BBC_PVN209 |
| 79 | BBC_PVN210 |
| 80 | BBC_PVN211 |
| 81 | BBC_PVN213 |
| 82 | BBC_PVN214 |
| 83 | BBC_ PVN 218 |
| 84 | BBC_PVN219 |
| 85 | BBC_PVN220 |
| 86 | BBC_PVN228 |
| 87 | BBC_PVN229 |
| 88 | BBC_PVN230 |
| 89 | BBC_PVN234 |
| 90 | BBC_PVN237 |
| 91 | BBC_PVN239 |
| 92 | BBC_PVN27 |
| 93 | BBC_PVN29 |
| 94 | BBC_PVN30 |
| 95 | BBC_PVN35 |
| 96 | BBC_PVN44 |
| 97 | BBC_PVN65 |
| 98 | BBC_PVN8 |
| 99 | BBC_PVN92 |
| 100 | BBC_PVN95 |
| 101 | BBC_PVN97 |
| 102 | BBC_ PVN B |
| 103 | BBC_Ref |
| 104 | BBC_ SB_ MUS1 |
| 105 | BBC_ SB_MUS2 |
| 106 | BBC_ SB_MUS3 |
| 107 | BBC_ SB_P54 |
| 108 | BBC_SB_P69 |
| 109 | BBC_ SB_PVN62 |
| 110 | BBC_ SB_PVN64 |
| 111 | BBC_SB_PVN66 |
| 112 | BBC_ SB_PVN67 |
| 113 | BBC_SB_PVN68 |
| 114 | BBC_ SB_PVN72 |
| 115 | BBC_ SB_PVN74 |
| 116 | BBC_SB_PVN7 |
| 117 | BBC_ SB_PVN81 |
| 118 | BBC_SB_PVN82 |
| 119 | BBC_T2 |

Table 2: Principal component scores as well as raw-material and level affiliations used for plots in the manuscript.

| PC1 | PC2 | Level | Raw Material |
| --- | --- | --- | --- |
| 0.040889 | -0.01419 | m1lo | silcrete |
| -0.0604 | -0.02659 | m1lo | silcrete |
| -0.03739 | -0.01113 | m1up | quartzite |
| 0.092433 | 0.027106 | m1lo | silcrete |
| -0.01405 | -0.02875 | m1up | quartzite |
| 0.032257 | 0.032018 | m1lo | silcrete |
| -0.0599 | -0.01757 | m1lo | silcrete |
| -0.02556 | 0.033566 | m1lo | silcrete |
| -0.07886 | -0.04219 | m1lo | quartzite |
| -0.00954 | 0.005398 | m1up | quartzite |
| 0.005029 | -0.00159 | m1lo | unknown |
| -0.05481 | 0.000314 | m1up | quartz |
| -0.04699 | -0.02037 | m1lo | silcrete |
| 0.015591 | -0.00479 | m1lo | silcrete |
| -0.00383 | 0.009509 | m1lo | quartz |
| -0.08242 | 0.008656 | m1up | unknown |
| 0.006762 | 0.020586 | m1up | silcrete |
| 0.016329 | 0.021956 | m1up | silcrete |
| -0.0578 | 0.005341 | m1up | silcrete |
| 0.036015 | 0.016002 | m1up | quartzite |
| -0.06341 | -0.01822 | m1up | silcrete |
| -0.02834 | 0.046449 | m1up | silcrete |
| -0.02399 | 0.016203 | m1up | quartzite |
| -0.00412 | -0.00031 | m1up | quartzite |
| -0.08464 | -0.01839 | m1up | silcrete |
| -0.01162 | -0.02901 | m1up | silcrete |
| -0.03299 | 0.009775 | m1up | silcrete |
| -0.02365 | -0.00808 | m1up | quartzite |
| -0.08048 | -0.01887 | m1up | quartzite |
| 0.082918 | -0.01605 | m1lo | silcrete |
| 0.094656 | 0.043026 | m1lo | silcrete |
| 0.100696 | 0.032452 | m1lo | silcrete |
| 0.066812 | 0.081638 | m1lo | silcrete |
| 0.09917 | 0.021634 | m1up | silcrete |
| -0.07111 | 0.001989 | m1lo | silcrete |
| 0.027536 | 0.008723 | m1lo | silcrete |
| -0.07043 | -0.00762 | m1up | quartz |
| 0.066596 | 0.006598 | m1lo | silcrete |
| -0.08013 | 0.010293 | m1up | quartz |
| 0.093692 | 0.033253 | m1lo | quartz |
| -0.06899 | -0.0095 | m1up | quartz |
| 0.040484 | 0.001029 | m1lo | silcrete |
| 0.005955 | 0.027656 | m1lo | silcrete |
| -0.06506 | -0.05525 | m1lo | silcrete |
| -0.0679 | -0.00059 | m1lo | quartzite |
| 0.025121 | 0.029572 | m1lo | quartz |
| -0.02057 | -0.03722 | m2 | quartzite |
| -0.03681 | -0.0134 | m1lo | silcrete |
| -0.02364 | 0.012537 | m1lo | silcrete |
| 0.086135 | -0.03625 | m2 | silcrete |
| -0.05318 | -0.06666 | m2 | silcrete |
| -0.00321 | 0.006756 | m1lo | silcrete |
| -0.01763 | 0.000847 | m1lo | silcrete |
| -0.01453 | -0.02348 | m1lo | silcrete |
| -0.00194 | 0.026179 | m1lo | quartzite |
| 0.001855 | -0.02975 | m2 | quartzite |
| -0.07383 | -0.04307 | m1up | silcrete |
| -0.06637 | -0.00473 | m1up | silcrete |
| 0.017847 | -0.02259 | m2 | silcrete |
| 0.017151 | -0.04905 | m1lo | silcrete |
| -0.00948 | -0.00299 | m1up | silcrete |
| 0.043048 | -0.01134 | m1lo | silcrete |
| 0.073348 | 0.033429 | m1up | silcrete |
| 0.106768 | 0.03233 | m1lo | silcrete |
| 0.095703 | 0.042569 | m1lo | silcrete |
| 0.044709 | 0.018609 | m1lo | silcrete |
| 0.055219 | 0.027261 | m1lo | silcrete |
| 0.050592 | -0.02767 | m2 | silcrete |
| 0.014456 | -0.0303 | m2 | silcrete |
| 0.086507 | 0.026595 | m1lo | silcrete |
| -0.03527 | -0.03482 | m1lo | quartz |
| 0.022608 | 0.004531 | m1lo | silcrete |

Bibliography

1. Villa P, Soressi M, Henshilwood CS, Mourre V. The Still Bay points of Blombos Cave (South Africa). Journal of Archaeological Science. 2009;36: 441–460. doi:10.1016/j.jas.2008.09.028
